# Variation in the *Neisseria lactamica* porin, and its relationship to meningococcal PorB

**DOI:** 10.1099/mic.0.2007/015479-0

**Published:** 2008-05

**Authors:** Julia S. Bennett, Martin J. Callaghan, Jeremy P. Derrick, Martin C. J. Maiden

**Affiliations:** 1 The Peter Medawar Building for Pathogen Research and Department of Zoology, University of Oxford, South Parks Road, Oxford OX1 3SY, UK; 2 Department of Paediatrics, University of Oxford, Centre for Clinical Vaccinology and Tropical Medicine, Churchill Hospital, Headington, Oxford OX3 7LJ, UK; 3 Manchester Interdisciplinary Biocentre, The University of Manchester, 131 Princess Street, Manchester M1 7DN, UK

## Abstract

One potential vaccine strategy in the fight against meningococcal disease involves the exploitation of outer-membrane components of *Neisseria lactamica*, a commensal bacterium closely related to the meningococcus, *Neisseria meningitidis*. Although *N. lactamica* shares many surface structures with the meningococcus, little is known about the antigenic diversity of this commensal bacterium or the antigenic relationships between *N. lactamica* and *N. meningitidis*. Here, the *N. lactamica* porin protein (Por) was examined and compared to the related PorB antigens of *N. meningitidis*, to investigate potential involvement in anti-meningococcal immunity. Relationships among porin sequences were determined using distance-based methods and *F_ST_*, and maximum-likelihood analyses were used to compare the selection pressures acting on the encoded proteins. These analyses demonstrated that the *N. lactamica* porin was less diverse than meningococcal PorB and although it was subject to positive selection, this was not as strong as the positive selection pressures acting on the meningococcal porin. In addition, the *N. lactamica* porin gene sequences and the protein sequences of the loop regions predicted to be exposed to the human immune system were dissimilar to the corresponding sequences in the meningococcus. This suggests that *N. lactamica* Por, contrary to previous suggestions, may have limited involvement in the development of natural immunity to meningococcal disease and might not be effective as a meningococcal vaccine component.

## INTRODUCTION

Meningococcal disease, caused by the Gram-negative bacterium *Neisseria meningitidis*, is a global health problem which cannot be comprehensively controlled by vaccination. Effective vaccines directed against the serogrouping antigen, the polysaccharide capsule, are available for disease caused by serogroups A, C, Y and W135 meningococci. However, a vaccine based on serogroup B polysaccharide has not been developed due to its poor immunological reactivity and similarity to host antigens (Finne *et al.*, 1987). As serogroup B strains cause over 85 % of meningococcal disease in the UK (Gray *et al.*, 2006), alternative vaccine candidates are under investigation, including outer-membrane proteins (OMPs), either purified (Boslego *et al.*, 1995) or as part of outer-membrane vesicle (OMV) formulations (Bjune *et al.*, 1991; van der Ley *et al.*, 1995).

The commensal bacterium *Neisseria lactamica,* which is closely related to both *N. meningitidis* and *Neisseria gonorrhoeae* (Guibourdenche *et al.*, 1986), is carried in the upper respiratory tracts of young children, in whom carriage of the meningococcus is rare. As natural immunity to meningococcal disease develops during childhood, carriage of *N. lactamica* may be involved in the acquisition of this immunity (Gold *et al.*, 1978). *N. lactamica* is only associated with disease in exceptional circumstances (Schifman & Ryan, 1983; Wilson & Overman, 1976) and as it does not possess a polysaccharide capsule (Griffiss *et al.*, 1987; Kim *et al.*, 1989) or the outer-membrane protein PorA (Ward *et al.*, 1992), this commensal or its antigens can be used in anti-meningococcal vaccines that are independent of both serogroup and serosubtype. Vaccines based on *N. lactamica* whole cells, OMPs, or OMVs have been proposed (Griffiss *et al.*, 1991; Oliver *et al.*, 2002) and research and development of a vaccine based on *N. lactamica* is ongoing (Finney *et al.*, 2007; Li *et al.*, 2006). However, the antigenic profiles of *N. lactamica* isolates are not well understood, and the cross-reactive epitopes that induce protection against meningococcal infection have not been defined (Tang *et al.*, 1999). This may constitute a major obstacle to the development of vaccines based on this species.

One protein that could be involved in the induction of cross-protective immune responses is the *N. lactamica* porin (Troncoso *et al.*, 2002). Porins, which are essential for growth, are pore-forming membrane proteins that create channels, allowing transport of hydrophilic molecules across lipid bilayers (Achouak *et al.*, 2001) and are generally highly expressed. These proteins exist as trimers (Derrick *et al.*, 1999) and consist of 16 anti-parallel *β*-strands connected by short periplasmic turns and eight surface-exposed loops (Maiden *et al.*, 1991; van der Ley *et al.*, 1991). The regions corresponding to the surface-exposed loops are less well conserved than the domains corresponding to the *β*-sheets, and show variation in both length and sequence, presumably as a consequence of immune selection (Maiden *et al.*, 1991; van der Ley *et al.*, 1991).

The *N. lactamica* porin is related to gonococcal PorB1a and PorB1b and to meningococcal PorB (Derrick *et al.*, 1999; Ward *et al.*, 1992) and is essentially identical to the *Neisseria polysaccharea* porin (Derrick *et al.*, 1999). Only these members of the genus contain three conserved lysine residues, found in close proximity within the pore, which form a potential GTP-binding site implicated in pathogenesis, consistent with a role in regulating pore function when inserted into host cells (Derrick *et al.*, 1999; Rudel *et al.*, 1996). Meningococcal PorB proteins are divided into two distinct, mutually exclusive classes designated PorB2 and PorB3. These proteins exhibit high levels of genetic diversity, especially in the surface-exposed regions, and these regions, with the exception of putative loops II and III, are subject to the diversifying influence of immunological selection (Urwin *et al.*, 2002). Putative loops II and III have structural roles; loop II is important in monomer–monomer interactions within the porin trimer, while loop III is sequestered in the pore of each monomer, and may be involved in regulating pore function (Derrick *et al.*, 1999).

Here, *N. lactamica por* sequences, in particular the regions encoding the putative surface-exposed loops, were examined and compared to *porB* of *N. meningitidis*, to investigate the diversity of the encoded proteins and to assess whether these *N. lactamica* proteins could be involved in the production of cross-protective immune responses to the meningococcus. This was achieved by comparing 69 unique *N. lactamica por* gene sequences, obtained from isolates previously characterized by multilocus sequence typing (MLST) (Bennett *et al.*, 2005) with a diverse collection of meningococcal *porB* sequences (Urwin *et al.*, 2002). Distance-based methods and *F_ST_* were used to determine relationships among the porins and maximum-likelihood analyses were used to investigate the selection pressures acting on these proteins in *N. lactamica*. The results indicated that the *N. lactamica* porin was only subject to weak positive selection pressures, had limited sequence similarity to meningococcal PorB and was less diverse than meningococcal PorB. This suggests that it might not be suitable for inclusion in anti-meningococcal vaccine formulations.

## METHODS

### Isolates.

A total of 587 individuals, including infants and a small number of siblings and parents, were sampled in Oxfordshire, UK, during two longitudinal studies of nasopharyngeal bacterial carriage in infants, resulting in 275 *N. lactamica* isolates. The genetic diversity of these *N. lactamica* isolates was similar to that seen in isolates from other locations and likely to be representative of the global diversity of this organism. There is no evidence that the Oxfordshire isolates were a separate population and the collection contained representatives from five of the six clonal complexes currently defined for *N. lactamica* (Bennett, 2006). Details of these isolates are available from the *Neisseria* MLST database: http://pubmlst.org/neisseria/ (Jolley *et al.*, 2004). Nucleotide sequences were determined for the entire *por* gene from all isolates. The majority had been collected from the same infants on successive occasions, enabling an analysis of the effect of carriage on the *N. lactamica* porin. The nucleotide and amino acid sequences from the *N. lactamica* porins were compared to the porin sequences from a diverse collection of 324 meningococcal isolates: http://neisseria.org/nm/typing/porb/ (Urwin *et al.*, 2002) which included sequences from both carried and disease-associated isolates.

### PCR amplification and sequence determination of *por* genes.

Amplification of the *por* gene was carried out by PCR with oligonucleotide primers B21 and B22 (Table 1). Amplification reaction mixtures contained reaction buffer [10 mM Tris/HCl (pH 8.3), 50 mM KCl, 1.5 mM MgCl_2_, 0.001 % (w/v) gelatin]; 200 μM each of dATP, dCTP, dGTP, dTTP; 1 μM of each primer; 1.25 units *Taq* polymerase (AmpliTaq; Applied Biosystems) per 50 μl; and 0.5 μl template DNA per 50 μl (approx. 50 ng μl^−1^). The PCR conditions consisted of an initial denaturation step of 94 °C for 2 min, followed by 35–40 cycles of denaturation (94 °C for 1 min), annealing (50–5 °C for 1 min), extension (72 °C for 2 min), and then a final extension step of 72 °C for 2 min. The PCR products were precipitated by incubation at room temperature for 30 min with 20 % (w/v) polyethylene glycol 8000, 2.5 M NaCl. After centrifugation for 60 min at 2750 ***g***, the precipitates were washed twice in 70 % (v/v) ethanol, dried and resuspended in 5–10 μl sterile distilled water.

The nucleotide sequences of the amplified gene fragments were determined at least once on each DNA strand by cycle sequencing with BigDye Ready Reaction Mix (Applied Biosystems), used in accordance with the manufacturer's instructions. Sequencing reactions were performed with the oligonucleotide primers listed in Table 1. Unincorporated dye terminators were removed by precipitation of the termination products with 95 % (v/v) ethanol and the labelled extension products were separated and detected with either a Prism 3700 DNA analyser, or a Prism 377 XL DNA analyser (Applied Biosystems).

### Analysis of sequence data.

Nucleotide sequence data from forward- and reverse-strand chromatograms were assembled into single contiguous sequences using the Staden suite of computer programs (Staden, 1996). Sequences were manually aligned using the SeqLab program, part of the GCG Wisconsin package, version 10.3 (Womble, 2000). The alignment (available on request) was based on amino acid sequence similarity, with codon integrity maintained. Each unique *por* allele was given a number in order of discovery, so that alleles were named *por-1* to *por-69*. To allow comparisons of *N. lactamica por* with the published meningococcal *porB* sequences (http://neisseria.org/nm/typing/porb/) (Urwin *et al.*, 2002), all sequences were truncated to the same length, so that the amino acid sequences began at the 32nd amino acid of the *N. lactamica* Por sequence (starting motif ETYRT) and ended with the 336th amino acid (motif STAST). mega version 2.1 (Kumar *et al.*, 2001) was used to calculate genetic distances between sequences and to produce neighbour-joining trees. To construct the tree from nucleotide sequences, all three coding positions were examined and the Kimura two-parameter distance correction (Kimura, 1980) was applied. A gamma shape parameter was not calculated as the inclusion of this parameter in the analysis had a minimal effect on the phylogeny produced. To produce the trees from amino acid sequences, *p*-distances were used. The reliability of the inferred trees was assessed using the bootstrap test (2000 replications). *F_ST_* values were calculated using Arlequin version 2.000 (Schneider *et al.*, 2000). Alignment gaps were excluded from all analyses.

### Analysis of selection pressures.

A maximum-likelihood approach was used to examine selection pressures acting on individual codon sites of the *N. lactamica por* gene. A phylogenetic tree of the aligned sequences was constructed using paup, version 4 (Swofford, 1998), using the HKY85 model of nucleotide substitution. Selection pressures on the aligned *por* sequences were examined using codeml, implemented in the paml program (Yang, 1997), in which codon substitution models were compared using the maximum-likelihood tree and the nucleotide sequence data. The *d*_N_/*d*_S_ ratio (parameter *ω*) was calculated codon by codon using different models of codon substitution that differed in how *ω* varied along the sequences. Model M0 estimated a single *ω* parameter for all sites, whereas the M1 model divided codons into two site categories: conserved sites (*p*_0_), with *ω*_0_ set at 0, and neutral sites (*p*_1_), with *ω*_1_ set at 1. To account for positive selection the M2 model was used, with the same two classes as M1 plus a third category of sites (*p*_2_), with *ω*_2_ estimated from the data. The M3 model was a more sensitive test of selection as it estimated *ω* values for three classes of sites, all of which could be >1. Models M7 and M8 both used a discrete beta distribution (with ten categories described by parameters *p* and *q*) to model *ω* ratios among sites, although M8 also included an additional class of sites for which *ω* could be >1.

Nested models were compared using a likelihood ratio test (LRT). Twice the difference in log-likelihood between models was compared with the value obtained under a *χ*^2^ distribution, with degrees of freedom equal to the difference in the number of parameters between models. Finally, empirical Bayesian methods were used to calculate the probability that a particular codon site belonged to a specific class.

## RESULTS

### Comparative diversity and relationships between *N. lactamica por* and meningococcal *porB* allele sequences

**Fig. 1. f1:**
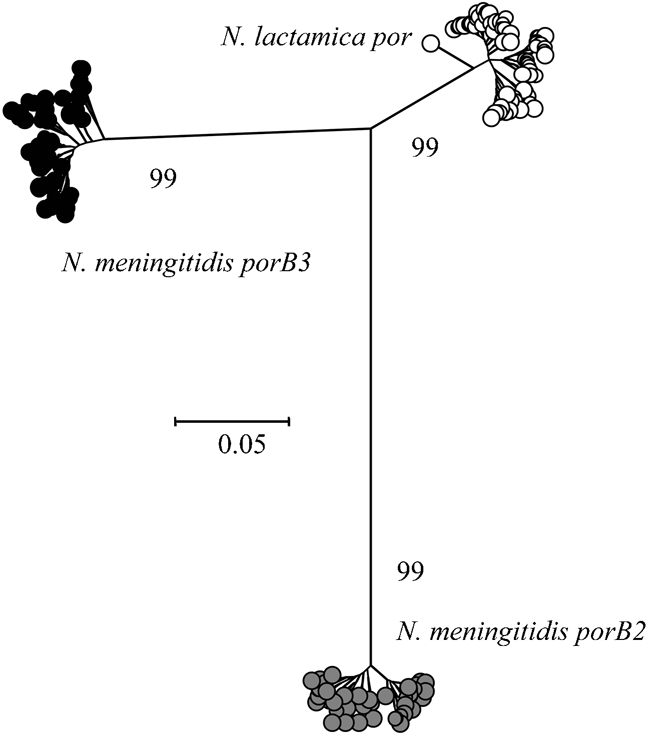
Neighbour-joining tree constructed from aligned gene sequences showing the relationship of *N. lactamica por* to meningococcal *porB2* and *porB3*. Bootstrap values are shown.

A total of 69 unique *N. lactamica por* alleles, obtained from 275 isolates, were aligned with 46 *porB*2 and 77 *porB*3 sequences, obtained from 324 diverse meningococcal isolates (Urwin *et al.*, 2002). A neighbour-joining tree was constructed from these sequences (Fig. 1), grouping the porin classes into three distinct clusters, supported by bootstrap values of 99 %. The *N. lactamica por* sequences were more closely related to *porB*3 than *porB*2, with less diversity than both meningococcal *porB* classes. *F_ST_* analysis of *por*, *porB2* and *porB3* was used to assess levels of gene flow among the populations and gave values >0.85 (*P*≤0.05) for all three comparisons, indicating separate populations with little genetic exchange among them. A neighbour-joining tree constructed from the nucleotide sequences from the *β*-barrel-encoding regions alone, using the same alignment, produced a tree topology almost identical to that shown in Fig. 1 (data not shown). The diversity within meningococcal *porB* and *N. lactamica por* alleles was assessed (Table 2). The number of variable sites was comparable, with 147 for *porB2*, 136 for *porB3* and 132 for *por*. Diversity, however, was greater within *porB2* and *porB3* (4.34 % and 4.03 % respectively) than in *por* (2.31 %). There were 44 unique amino acid sequences for PorB2, 65 for PorB3 and 62 for Por, with more variable sites within PorB2 (70) and PorB3 (61) than in Por (49).

### Comparative diversity and relationships between *N. lactamica* Por and meningococcal PorB loop regions

The amino acid sequence variants of the putative Por loop regions were named in accordance with the meningococcal PorB nomenclature used in the PorB database (http://neisseria.org/nm/typing/porb/), although the location and length of the loops were as defined previously (Derrick *et al.*, 1999). Six variants were found in loop I, eight in loop II, 17 in loop III, four in loop IV, 10 in loop V, five in loop VI, and three in loops VII and VIII. The loops were of variable length (11 amino acids in loops II, IV and VI, 13 in loop VII, 16 in loop VIII, 18 in loop V, 23 in loop I and 41 in loop III). Some of the amino acid sequences were encoded by more than one nucleotide sequence, and one of the variants found in loop III was longer than the other variants by two dipeptides: ST and GI. The eight loop regions in *N. lactamica* Por were compared to the corresponding loops in meningococcal PorB (Table 2). Mean percentage *p*-distances within the loops of Por, PorB2 and PorB3 were determined, and for each loop, with the exception of loops II and III, divergence was greater within PorB2 and PorB3 than within Por.

**Fig. 2. f2:**
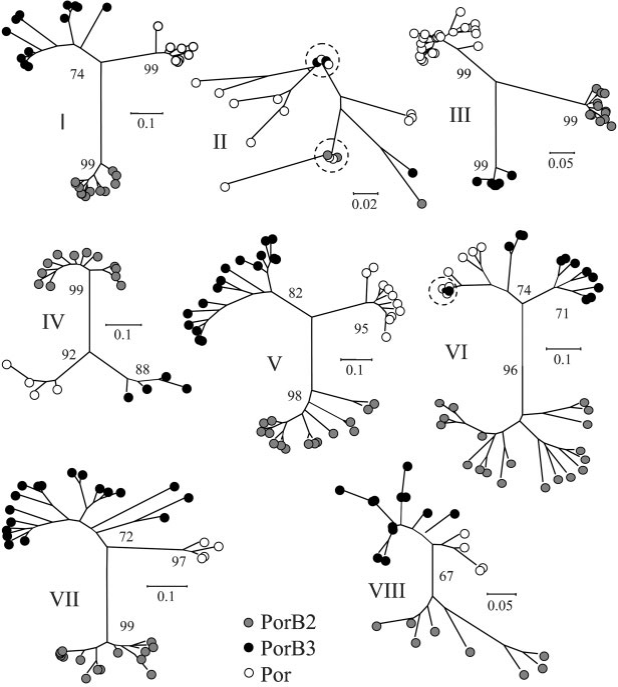
Neighbour-joining trees constructed from the aligned amino acid sequences from the loop regions of *N. lactamica* Por and meningococcal PorB2 and PorB3. Amino acid sequences identical in *N. lactamica* and *N. meningitidis* are indicated by dashed circles. Bootstrap values are shown.

Neighbour-joining trees were constructed from the amino acid sequences of the loop regions from Por, PorB2 and PorB3 (Fig. 2). For the majority of the loops, distinct clusters, corresponding to the three different classes, were formed. In loop II the groups were indistinct and not well supported by bootstrap values (not included), with two PorB2 and two PorB3 amino acid sequences identical to Por sequences from *N. lactamica*. One of these PorB2 sequences was encoded by a nucleotide sequence identical to that of *N. lactamica*. Three distinct clusters were evident for loop III, with this loop more uniform in the meningococcal porins than in the *N. lactamica* porin. In loop VI the PorB2 variants formed a distinct group but the PorB3 variants were more closely related to *N. lactamica* Por. A loop VI PorB3 amino acid sequence was identical to the amino acid sequences from three *N. lactamica* alleles, and clustered with the *N. lactamica* sequences. This PorB3 variant was encoded by a nucleotide sequence identical to one of the *N. lactamica* variants.

### Maximum-likelihood analysis

Maximum-likelihood analysis provided evidence for positive selection pressures acting on the *por* gene of *N. lactamica* (Table 3), as models with site classes where parameter *ω* was permitted to be >1 (models M2, M3 and M8) were statistically significantly better than those in which it was not (models M0, M1, M7). However, the M3 model (a sensitive test of positive selection) was not significantly better supported than the more conservative M2 model (*P*=0.251). Under the M2 model, 7.0 % of sites fell into a positively selected class, where *ω*_2_=4.195. Under model M3, 7.1 % of sites fell into a positively selected class, where *ω*_2_=3.936, 8.5 % of sites fell into a neutrally evolving class where *ω*_1_=1.002 and the remaining 84.4 % of sites were highly conserved (*ω*_0_=0.013). Under model M8 7.3 % of sites fell into a positively selected class, where *ω*_1_=3.886.

**Fig. 3. f3:**
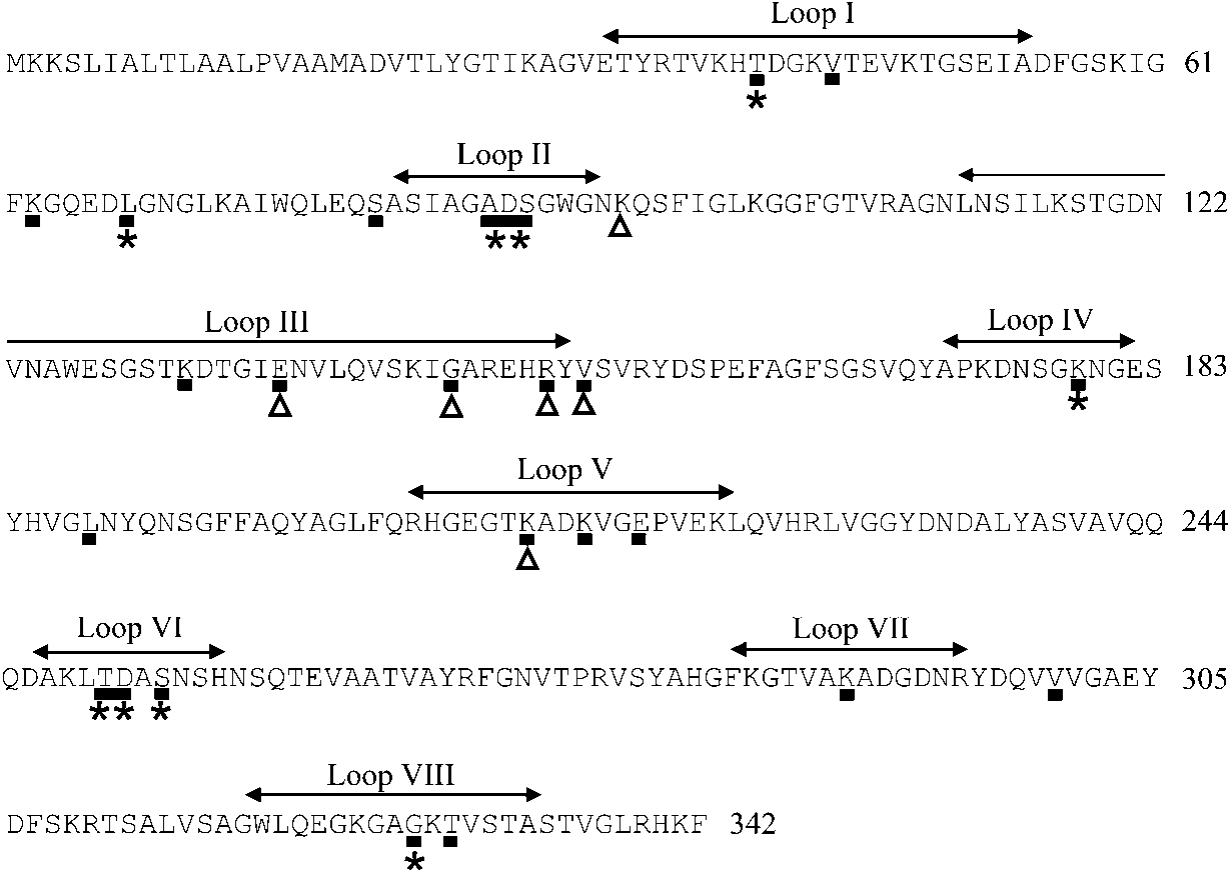
Positive selection acting on *N. lactamica* porin sequences. The translated sequence of *por-2*, one of the longest sequences, is used as an example. The locations of the putative loop regions (loops I–VIII) are indicated. Amino acid residues subject to positive selection under models M2 and M8 are identified with asterisks. Amino acid residues subject to positive selection under the M3 model are indicated with black boxes. Polymorphic sites in the porin alleles of the genotypically identical (ST-608) isolates carried by one infant are indicated by triangles. Figures at the end of each line of sequence denote residue number.

Bayesian methods were used to identify the sites with the highest probability of falling into the positively selected class under models M2, M3 and M8. A *d*_N_/*d*_S_ ratio (parameter *ω*) >1.5 was considered to be indicative of positive selection, and for model M2, there were nine selected sites with *d*_N_/*d*_S_ ratios of 4.068–4.195. The same nine sites were identified using model M8, although the *d*_N_/*d*_S_ ratios were 3.825–3.886. Model M3, which had previously been used to demonstrate selection in meningococcal PorB (Urwin *et al.*, 2002) and a more sensitive test of selection than model M2, identified 16 additional selected sites with *d*_N_/*d*_S_ ratios of 1.900–3.936. The majority of selected sites fell within predicted loop regions, with positive selection identified in loops I, II, IV, VI and VIII using all three models. Only model M3 identified selected sites within loops III, V and VII. All selected sites were mapped onto the translated sequence of *por-2* (Fig. 3).

### A *N. lactamica* porin structural model

**Fig. 4. f4:**
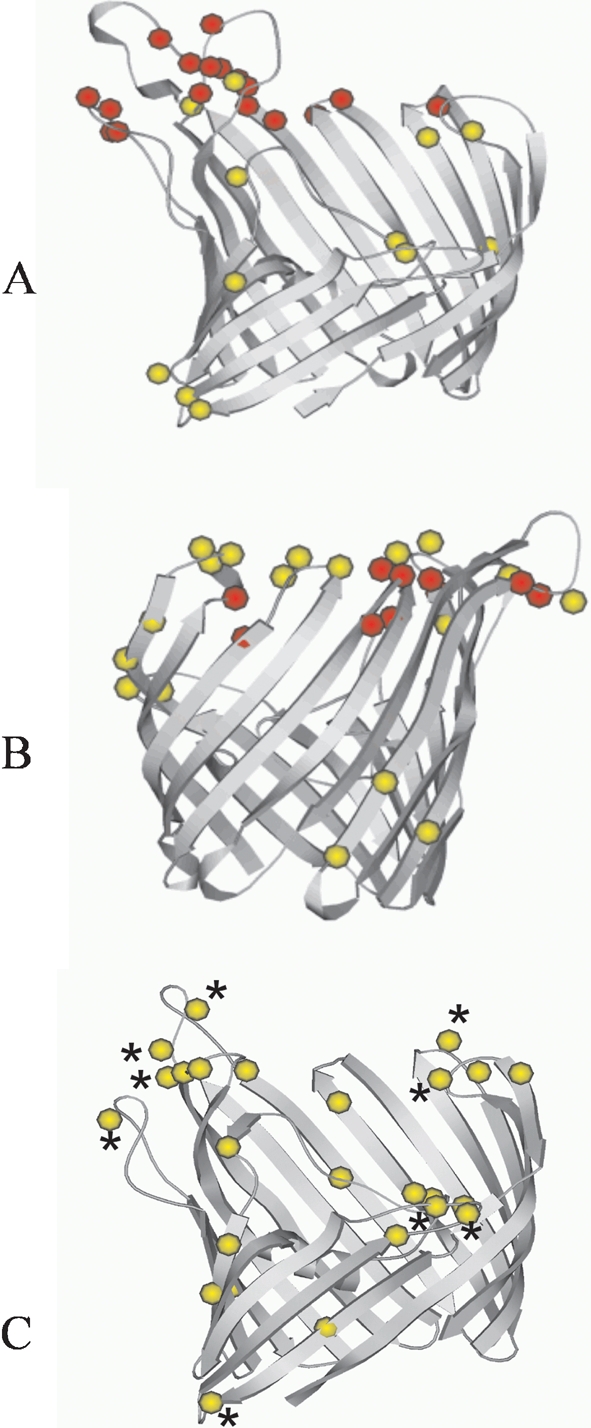
Ribbon diagrams of models for meningococcal PorB2-5 (A) PorB3-2 (B) and *N. lactamica* Por-2 (C), with superposition of residues subject to positive selection. Residues under strong selection are shown as red spheres and residues under weaker selection are shown as yellow spheres, under model M3. Residues subject to positive selection under models M2 and M8 are identified with asterisks. Some longer loop regions have been truncated. Diagrams produced using Molscript (Kraulis, 1991).

A homology model for the *N. lactamica* porin was constructed in the same way as corresponding models for meningococcal PorB2 and PorB3 (Urwin *et al.*, 2002). The translated sequence of *N. lactamica por-2* was aligned against that of the porin Omp32 from *Comamonas acidovorans* (Zeth *et al.*, 2000), which was used as a template for structural alignment and refinement, as implemented by the software package modeller (Sali *et al.*, 1995). The three-dimensional structural model of the *N. lactamica* porin is shown along with the published models of meningococcal PorB2 and PorB3 (Urwin *et al.*, 2002) in Fig. 4. Positively selected sites, as inferred from maximum-likelihood analysis using model M3, were mapped onto this model, with model M2 and model M8 positively selected sites highlighted. Whereas a number of strongly selected sites were located within the surface-exposed loops for both meningococcal PorB2 and PorB3, only weakly positively selected sites were present in the *N. lactamica* porin structure. In the *N. lactamica* porin, using model M3, weakly positively selected sites were evident within the pore. Weakly positively selected sites were also present within the pore of meningococcal PorB2, but not PorB3. The general structure of all three porins was similar, although the *N. lactamica* porin was closer to meningococcal PorB3, as some of the surface-exposed loops were shorter in both Por and PorB3.

### Selection pressures acting on *N. lactamica* porins during carriage

As the majority of *N. lactamica* isolates were collected from the same infants sampled on successive occasions, it was possible to examine the effects of carriage on the porins of these sequential isolates. There was long-term carriage of particular variants, with little evidence for replacement of variants. One child carried isolates with the MLST genotype ST-638 on seven successive samplings, between the ages of 12 weeks and 48 weeks, but at age 48 weeks the porin allele associated with these isolates altered by one nonsynonymous nucleotide substitution in loop VI. This allele was not present among the isolates obtained from any other subjects and is likely to have arisen by point mutation as a result of immune selection during carriage by this particular child. This site was also variable in other *N. lactamica* porin alleles (position 253, Fig. 3), with a *d*_N_/*d*_S_ value >3.860 using all three models of selection.

Another infant carried ST-608 isolates on eight successive occasions, from age 8 weeks until age 96 weeks, but seven different porin alleles were identified among these isolates. There were six polymorphic sites among these alleles (Fig. 3), with each change nonsynonymous, altering the structure of the porin. Most were found within and around loop III, which potentially influences pore function. One was found within loop V, which may have occurred as a result of immune selection. These sites, except for position 95, were also variable in other *N. lactamica* porins. However, only model M3 detected positive selection at these sites with *d*_N_/*d*_S_ values of ∼3.648, except for position 95 (*d*_N_/*d*_S_ 1.487).

## DISCUSSION

Establishing the diversity of antigen genes from non-pathogenic neisseriae is important, as antigens common to *N. meningitidis*, *N. lactamica* and other non-pathogenic commensals may be involved in conferring natural immunity to meningococcal disease (Troncoso *et al.*, 2000, 2002). *N. lactamica* antigens are also components of anti-meningococcal vaccines currently being developed (Gorringe, 2005) and the identity and characterization of the key cross-reactive antigenic components is of great importance (Finney *et al.*, 2007). Although the porins of *N. meningitidis* have been thoroughly examined (Russell *et al.*, 2004; Urwin *et al.*, 2002), little information was available on the diversity of the *N. lactamica* porin and its relationship to meningococcal porins (Derrick *et al.*, 1999; Ward *et al.*, 1992). Here, the diversity of 69 *N. lactamica por* alleles was determined and compared to the related meningococcal *porB* alleles to assess the potential for antigenic cross-reactivity.

The neighbour-joining tree constructed from *N. lactamica por* and meningococcal *porB2* and *porB3* alleles demonstrated the relatively low diversity among the *N. lactamica por* alleles and established that these alleles were distinct from *porB2* and *porB3*. The distinct clusters observed suggest that genetic exchange between *N. lactamica por* and the two meningococcal *porB* classes is likely to be rare. This was supported by the *F_ST_* results, which indicated low levels of gene flow between the three groups. The neighbour-joining tree constructed from the sequences that encode the conserved *β*-barrel regions alone, which were subject to stabilizing selection, produced a tree topology almost identical to the tree constructed from the sequences that encode both the *β*-barrel regions and the loop regions. This suggests that the porins have not diverged recently.

The amino acid sequences of the surface-exposed loop regions were analysed individually and the loops that determine antigenic variability in *N. meningitidis* (loops I, IV, V, VI, VII and VIII) (Derrick *et al.*, 1999) were dissimilar to the corresponding loops in *N. lactamica*. Loop II sequences were the most similar between *N. lactamica* and *N. meningitidis*, and sequences from two PorB2 and two PorB3 loop II variants were identical to loop II *N. lactamica* variants. As structural constraints are likely to limit diversity in this region (Derrick *et al.*, 1999), these identical sequences are probably a consequence of shared ancestry rather than recent lateral genetic transfer. This is supported by the detection of minimal gene flow between these three populations. A single example of lateral genetic transfer between species may have occurred in loop VI. This loop was subject to positive immune selection and a meningococcal PorB3 variant was encoded by a nucleotide sequence identical to a *N. lactamica* sequence. However, two other *N. lactamica* variants had amino acid sequences identical to the PorB3 variant but were encoded by different nucleotide sequences.

Frequent genetic recombination between species has been described for *tbpB* (Linz *et al.*, 2000) and may also occur within the variable region of *fetA* (J. S. Bennett, E. A. L. Thompson, P. Kriz, K. A. Jolley & M. C. J. Maiden, unpublished results). However, genetic exchange frequency decreases rapidly as a function of relatedness of the donor to the recipient (Majewski, 2001). As the *N. lactamica* porin is distinct from the meningococcal porins, including PorA (Derrick *et al.*, 1999), and does not experience strong selection pressures, frequent lateral genetic transfer of porin sequences between the two species is unlikely, in common with the housekeeping genes of *Neisseria* (Bennett *et al.*, 2007).

Porins, including those of *Neisseria*, have an internal eyelet region formed by a long loop III folded into the pore, with a negatively charged cluster of side-chains facing the positive charges from R/K residues derived from *β*-sheets forming the *β*-barrel wall (Achouak *et al.*, 2001; Schirmer, 1998). This organization produces an electrostatic field in the lumen that regulates the diffusion of molecules through the constricted area (Achouak *et al.*, 2001). Loop III was more variable in *N. lactamica* Por than in meningococcal PorB. The amino acid sequences encoded by *por-2* and *por-8* were the longest *N. lactamica* sequences due to two dipeptide insertions (S T and G I), occurring at the tip of loop III. As this loop folds back into the centre of the lumen, these insertions could affect pore conductance. The sequence motif found in this region of the translated sequences of *por*-2 and *por*-8 (STKDTGI) was not present in any other *N. lactamica* or meningococcal sequence examined in this study and may have been transferred from another bacterial species by lateral genetic transfer.

Analysis of the selection pressures acting on the *N. lactamica* porin revealed codons subject to positive selection, the majority of which were located within the loop regions. However, due to the variability of these regions in both *N. meningitidis* and *N. lactamica* and the difficulty in aligning the amino acid sequences unambiguously, it was not possible to determine if the positively selected codons in *N. lactamica* were the same as those positively selected in *N. meningitidis*. Whereas some residues in meningococcal PorB2 and PorB3 were under strong positive immune selection, with *d*_N_/*d*_S_ ratios (*ω*) of 18.553 for PorB2 and 13.923 for PorB3 (Urwin *et al.*, 2002), residues in the *N. lactamica* porin experienced much weaker positive selective pressures, with *d*_N_/*d*_S_ ratios that did not exceed 4.195.

Results from the three maximum-likelihood models that took account of positive selection agreed on nine positively selected codon sites in the *N. lactamica* porin. The M3 model identified an additional 16 positively selected sites. These additional sites were considered in this analysis as five of them were polymorphic in isolates with identical MLST genotypes that were carried by one infant on successive occasions. A sixth site was only polymorphic in the porin of the isolates carried by this particular infant, although the *d*_N_/*d*_S_ value for this site (1.487) was less than the cut-off value for positive selection (>1.5) used in this analysis. Both the M2 and M8 models failed to detect positive selection at these sites which were biologically relevant as the changes were nonsynonymous, altering the structure of the porin.

Using model M3, three weakly positively selected codon sites were detected in loop II of the *N. lactamica* porin, whereas in the meningococcus, only one weakly positively selected site was present in loop II of PorB3 and none in loop II of PorB2 (Urwin *et al.*, 2002). This suggests that there is less structural constraint in this region in *N. lactamica* than in the meningococcus. Under model M3, four weakly positively selected sites were detected in loop III of the *N. lactamica* porin, three of which were variable among the sequential isolates with identical MLST genotypes carried by one infant. As loop III is sequestered within the lumen, these variations may reflect changes in pore function. Three weakly positively selected sites were present in loop III of meningococcal PorB2 but none in loop III of PorB3 (Urwin *et al.*, 2002), suggesting that *N. lactamica* Por is more like PorB2 in this respect. *N. lactamica* has a single porin as opposed to the two in most meningococci, and variations in the permeability of the pore might alter the function of this protein, improving fitness. In the gonococcus, which also has one functional porin (Feavers & Maiden, 1998), mutations in loop III were shown to reduce porin permeability to hydrophilic antibiotics and these changes may therefore have a role in resistance to penicillin and tetracycline (Gill *et al.*, 1998). However, the genotypically identical *N. lactamica* isolates that expressed seven different *por* alleles while carried by a single infant had not been exposed to antibiotic therapy (D.W. Crook, personal communication) and therefore the reasons for the rapid amino acid variation among these isolates remain unexplained.

Although the *N. lactamica* porin undergoes relatively weak positive selection pressures in comparison to the meningococcus, nonsynonymous changes in the porin alleles among isolates carried by two infants showed plasticity of the porin, probably in response to selection, over a very short time period. This supports previous studies that established that meningococcal porins are subject to strong positive selection pressures and evolve rapidly (Jelfs *et al.*, 2000; Urwin *et al.*, 2002), hindering the design of vaccines based on these proteins.

Initially, *N. lactamica por* appeared to be a good candidate for involvement in the production of a cross-protective immune response to meningococci, as the related meningococcal PorB protein is subject to strong immune selection (Urwin *et al.*, 2002). Porins are also essential for bacterial growth and are expressed at high levels, and *por* genes were amplified from all *N. lactamica* isolates examined. However, the *N. lactamica* porin was dissimilar to the porins of *N. meningitidis,* with no alleles shared, and the surface-exposed loop region amino acid sequences were rarely identical in meningococci and *N. lactamica*. The low sequence identity between Por and PorB, the low levels of genetic exchange, as indicated by the *F_ST_* results, together with the relatively weak positive selection pressures acting on the *N. lactamica* porin and the lack of immunological cross-reactivity with meningococcal PorB (Finney *et al.*, 2007; Kim *et al.*, 1989), suggest that this protein may not be important in the induction of any cross-protective immune responses that might protect against meningococcal disease. Therefore, any protection against meningococcal disease provided by *N. lactamica* carriage is likely to be due to antigens other than Por.

## Figures and Tables

**Table 1. t1:** Oligonucleotide primers used in *N. lactamica* *por* sequencing

**Primer**	**Sequence (5′–3′)**	**Function**
B21 (forward)*	CCAAAAAAGGAATACAGC	Amplification and sequencing
B22 (reverse)*	GCAGATTAGAATTTGTGG	Amplification and sequencing
8U (forward)†	TCCGTACGCTACGATTCTCC	Sequencing
8L (reverse)†	GGAGAATCGTAGCGTACGGA	Sequencing
94U (forward)†	CTCAAACCGAAGTTGCCG	Sequencing
94L (reverse)†	CGGCAACTTCGGTTTGAG	Sequencing

*Ward *et al.* (1992). †Suker (1997).

**Table 2. t2:** Diversity within meningococcal *porB* and *N. lactamica por* genes

	***porB2***	***porB3***	***por***
No. of alleles	46	77	69
Length of aligned sequences (bp)	1053	894	1029
No. of variable sites	147	136	132
Mean percentage genetic distance (Kimura's two-parameter model)	4.34	4.03	2.31
	**PorB2**	**PorB3**	**Por**
No. of amino acid sequences	44	65	62
No. of variable sites	70	61	49
Mean percentage *p*-distance			
Loop I	10.60	24.80	8.39
Loop II	6.06	6.06	12.00
Loop III	4.89	2.27	6.87
Loop IV	15.23	13.89	12.12
Loop V	24.51	30.67	13.77
Loop VI	32.78	28.86	14.72
Loop VII	15.71	34.84	7.69
Loop VIII	24.11	11.93	7.29

**Table 3. t3:** Parameter estimates for the maximum-likelihood analysis of selection pressures acting on the *N. lactamica* porin

**Model**	**Site categories (*p*) and *d_N_ / d_S_* (*ω*)**	**Likelihood test**	***χ*^2^**	***P***
M0	*ω*=0.306			
M1	*p*_0_=0.816, *p*_1_=0.184			
M2	*p*_0_=0.818, *p*_1_=0.116, *p*_2_=0.070	M0 vs M2	387.144	<0.000
	*ω*_2_=4.195	M1 vs M2	70.442	<0.000
M3	*p*_0_=0.844, *p*_1_=0.085, *p*_2_=0.071	M0 vs M3	389.907	<0.000
	*ω*_0_=0.013, *ω*_1_=1.002, *ω*_2_=3.936	M1 vs M3	73.205	<0.000
		M2 vs M3	2.763	0.251
M7	*P*=0.026, *q*=0.118			
M8	*P*=0.035, *q*=0.330	M7 vs M8	72.475	<0.000
	*p*_1_=0.073, *ω*_1_=3.886			

## Abbreviations

- MLST, multilocus sequence typing
- OMP, outer-membrane protein
- OMV, outer membrane vesicle
